# The basics of preclinical drug development for neurodegenerative disease indications

**DOI:** 10.1186/1471-2377-9-S1-S2

**Published:** 2009-06-12

**Authors:** Karen L Steinmetz, Edward G Spack

**Affiliations:** 1 Biosciences Division, SRI International, 333 Ravenswood Ave, Menlo Park, CA 94025, USA

## Abstract

Preclinical development encompasses the activities that link drug discovery in the laboratory to initiation of human clinical trials. Preclinical studies can be designed to identify a lead candidate from several hits; develop the best procedure for new drug scale-up; select the best formulation; determine the route, frequency, and duration of exposure; and ultimately support the intended clinical trial design. The details of each preclinical development package can vary, but all have some common features. Rodent and nonrodent mammalian models are used to delineate the pharmacokinetic profile and general safety, as well as to identify toxicity patterns. One or more species may be used to determine the drug's mean residence time in the body, which depends on inherent absorption, distribution, metabolism, and excretion properties. For drugs intended to treat Alzheimer's disease or other brain-targeted diseases, the ability of a drug to cross the blood brain barrier may be a key issue. Toxicology and safety studies identify potential target organs for adverse effects and define the Therapeutic Index to set the initial starting doses in clinical trials. Pivotal preclinical safety studies generally require regulatory oversight as defined by US Food and Drug Administration (FDA) Good Laboratory Practices and international guidelines, including the International Conference on Harmonisation. Concurrent preclinical development activities include developing the Clinical Plan and preparing the new drug product, including the associated documentation to meet stringent FDA Good Manufacturing Practices regulatory guidelines. A wide range of commercial and government contract options are available for investigators seeking to advance their candidate(s). Government programs such as the Small Business Innovative Research and Small Business Technology Transfer grants and the National Institutes of Health Rapid Access to Interventional Development Pilot Program provide funding and services to assist applicants in preparing the preclinical programs and documentation for their drugs. Increasingly, private foundations are also funding preclinical work. Close interaction with the FDA, including a meeting to prepare for submission of an Investigational New Drug application, is critical to ensure that the preclinical development package properly supports the planned phase I clinical trial.

## Introduction

The drug development process is typically divided into three major steps: discovery, preclinical development, and clinical trial. The transition from discovery to preclinical development is a continuum, and results of preliminary pharmacology and toxicology testing often contribute to lead drug candidate selection. The boundary between preclinical development and clinical trial is sharply defined by the filing of an Investigational New Drug (IND; Table 1 lists preclinical development acronyms) application, which is required prior to initiation of the clinical trial. The activities supporting an IND application are the subject of this overview. The adage 'begin with the end in mind' is particularly appropriate for preclinical development, as the resulting IND must support the planned clinical trial design. For example, a clinical trial involving daily chronic administration requires repeat-dose toxicity studies in preclinical animal models.

**Table 1:** Preclinical development acronyms

ADME	Absorption, distribution, metabolism, and excretion
API	Active pharmaceutical ingredient: any component intended to furnish pharmacological activity or other direct effect in the diagnosis, cure, mitigation, treatment or prevention of disease.
CFR	Code of Federal Regulations
CGMP, GMP	(Current) good manufacturing practice
CMC	Chemistry manufacturing and controls
CoA	Certificate of analysis
CRO	Contract research organization
CTM	Clinical trial material
c_max_	Maximum plasma concentration
DP	Drug product: finished dosage form (for example, tablet, capsule, solution) that contains an active drug ingredient, generally in association with inactive ingredients
DS	Drug substance: any substance that is represented for use in a drug and that, when used in manufacturing, processing, or packaging of a drug, becomes an active ingredient or a finished drug form
FDA	US Food and Drug Administration
FIH	First in human
FRS	Foreign related substances
GLP	Good laboratory practice
HPLC	High performance liquid chromatography
ICH	International Conference on Harmonisation
IND	Investigational New Drug application
MTD	Maximum tolerated dose
NCE	New chemical entity
NDA	New drug application
NCI	National Cancer Institute
NIA	National Institute on Aging
NOAEL	No observed adverse effect level
PIB	Powder in bottle
PK	Pharmacokinetics
RAID	Rapid Access to Intervention Development (preclinical program)
SBIR	Small Business Innovative Research (grant)
STTR	Small Business Technology Transfer (grant)
TI	Therapeutic Index
TK	Toxicokinetic
t_max_	Time of maximum plasma concentration after dose administration
TTP	Target product profile

**Figure 1:**
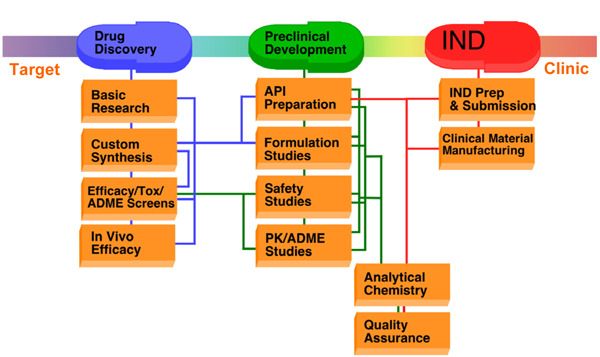
**Preclinical drug development stages**. Following identification of a drug target and candidate compounds, several early activities, such as pharmacology, *in vivo *efficacy, and experimental toxicology, can contribute to the selection of a lead candidate for preclinical development. These preclinical activities provide the basis for an Investigational New Drug (IND) application to the FDA for permission to initiate clinical testing in humans. ADME, absorption, distribution, metabolism, and excretion; API, active pharmaceutical ingredient; PK, pharmacokinetics; Prep, preparation; Tox, toxicity.

**Figure 2:**
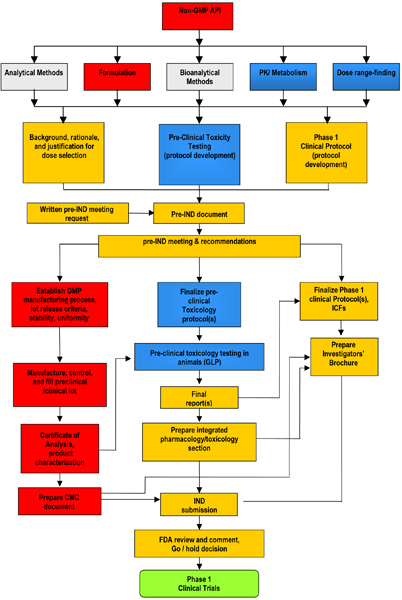
**Preclinical flow diagram**. The parallel and inter-related activities contributing to preclinical development are summarized with color coding to denote related components: manufacturing (red), analytical (grey), documentation (orange), safety (blue), clinical (green). API, active pharmaceutical ingredient; CMC, chemistry, manufacturing, and controls; FDA, US Food and Drug Administration; GLP, good laboratory practice; GMP, good manufacturing practice; ICF, informed consent form; IND, Investigational New Drug; PK, pharmacokinetics.

Once a lead candidate is identified, a typical preclinical development program consists of six major efforts: manufacture of drug substance (DS)/active pharmaceutical ingredient (API); preformulation and formulation (dosage design); analytical and bioanalytical methods development and validation; metabolism and pharmacokinetics; toxicology, both safety and genetic toxicology and possibly safety pharmacology; and good manufacturing practice (GMP) manufacture and documentation of drug product for use in clinical trials (Figure 1) The IND application summarizes the results of the above activities for submission to the US Food and Drug Administration (FDA). Table 2 outlines the general organization of an IND application and lists many of the relevant Code of Federal Regulations (CFR) sections for each key component. These activities are seldom discrete and sequential; rather, they are interrelated and often concurrent, with results from each activity informing the other steps as the drug candidate progresses through characterization and optimization (Figure 2).

**Table 2:** IND application table of contents

1	Form FDA 1571	[21 CFR 312.23(a)(1)]
2	Table of contents	[21 CFR 312.23(a)(2)]
3	Introductory statement	[21 CFR 312.23(a)(3)]
4	General investigational plan	[21 CFR 312.23(a)(3)]
5	Investigator's brochure	[21 CFR 312.23(a)(5)]
6	Protocol(s)	[21 CFR 312.23(a)(6)]
	a. Study protocols	[21 CFR 312.23(a)(6)]
	b. Investigator data	[21 CFR 312.23(a)(6)(iii)(b)]^a^
	c. Institutional review board data	[21 CFR 312.23(a)(6)(iii)(b)]^a^
7	Chemistry, manufacturing, and control data	[21 CFR 312.23(a)(7)]
8	Pharmacology and toxicology data	[21 CFR 312.23(a)(8)]
9	Previous human experience	[21 CFR 312.23(a)(8)]
10	Additional information	[21 CFR 312.23(a)(10)]

^a^Or completed form(s) FDA 1572.

## Preclinical development components

### Target product profile

Stephen Covey's advice to 'begin with the end in mind' from his bestseller *The 7 Habits of Highly Effective People *[1] is also applicable to drug development. The goal in drug development is FDA approval of a new drug application (NDA) and prescribed use in the clinic. Many project development teams find it helpful to develop a target product profile (TPP) to guide preclinical development. The TPP is a useful tool for delineating the required as well as desired features of the new drug product, critical milestones, and metrics to success. The TPP provides a framework to ensure that the preclinical development program supports the intended clinical trial design and therapeutic use [2]. The contents of a TPP may vary from compound to compound and from team to team, but each profile generally includes therapeutic indication(s); market size, competition, and differentiators; expected clinical use, including key trial endpoints; drug target and mechanism of action; patient age range; dose route, form, and frequency of administration; bioavailability and duration of action; safety, precautions, and contraindications; chemistry, manufacturing, and controls (CMC) profile, including solubility, manufacturing process, formulation, storage conditions, and stability; patent status and any modifiers of exclusivity (for example, orphan drug status).

In the case of chronic neurodegenerative diseases such as Alzheimer's disease, the unique medical needs of elderly patients have the potential to affect several aspects of drug design. Alleviation of Alzheimer's disease will most likely require long-term treatment and, therefore, the preclinical toxicology program must include repeat-dose administration to mimic the dosing regimen expected in the clinic. Since Alzheimer's patients are generally well past child-bearing age, some delay in safety testing for genetic and reproductive toxicity potential is permitted. On the other hand, it may be important to evaluate the potential for drug-drug interaction much earlier in the drug development program since many elderly patients are likely to be prescribed medications to manage blood pressure, cardiovascular diseases, metabolic and digestive disorders, joint inflammation, diabetes, and other conditions associated with aging. Project teams may select a route and frequency of dose administration that is optimal for compliance in elderly Alzheimer's disease patients. For example, an oral dose formulation composed of either small capsules/tablets or a liquid to be administered once daily or less frequently may be included in the TPP as a required product feature. Many drugs targeting neurodegeneration such as Alzheimer's disease will need to cross the blood brain barrier to access the cellular target. Depending on the cellular mechanism, bioavailability, particularly to the brain, at or above a selected target concentration may be another required feature delineated in the TPP.

The TPP document continues to evolve as the drug development program progresses, and it should be regularly reviewed by the team to assess whether required goals are being met. Any results indicating potential safety concerns or the inability of a given new chemical entity to fulfill stated TPP criteria should be evaluated by the team for their potential impact on project success. Implementation of a TPP helps to keep the focus of the project on key product criteria, thereby increasing the likelihood that 'Go/No-Go' decisions will be made in a timely manner and reducing the overall project risk.

### Pharmacology and experimental toxicology

The prelude to IND-enabling preclinical development generally includes efficacy, pharmacology, and experimental toxicology studies to define the dose, route, and frequency required for subsequent studies. Using one or more pharmacological animal models of the disease, the initial efficacy studies demonstrate that treatment with drug candidates has the desired therapeutic effect. Efficacy studies also help to identify the best drug candidates for further development. A number of studies are used to address the absorption, distribution, metabolism, and excretion (ADME) characteristics of the drug. Bioavailability studies are generally conducted *in vivo *on candidates intended to be administered by a nonintravenous route. Bioavailability results provide information on the percentage of drug that is absorbed by the body as defined by quantity in plasma. Pharmacokinetics (PK) studies provide information on the maximum attainable plasma concentration (c_max_), the time after dose administration to c_max _(t_max_), the mean residence time in the plasma, clearance, and other information used to characterize the body's effect on the drug.

Initial dose range-finding and toxicity studies include single- and multiple-dose administration protocols with varied observation times. These earliest studies are intended to determine the maximum tolerated dose (MTD), identify observable signs of toxicity, and provide a rationale for setting dose levels in more complex definitive studies. Regulatory requirements almost always call for definitive studies in at least two laboratory animal species, one rodent (rat or mouse) and one nonrodent (rabbit, dog, nonhuman primate, or other suitable species). Preliminary toxicity, bioavailability, and PK studies should also include one or more rodent and nonrodent species, including the species to be used in the definitive studies. The group sizes for the early range-finding studies may consist of only a few animals and one sex (one animal per dose level). Once a suitable dose range is identified, group sizes are increased to at least three per sex per dose level to allow for statistical comparisons. Only a few endpoints are collected in these range-finding studies unless there are particular toxicity concerns; a larger number of required endpoints will be evaluated in the eventual definitive studies described below. Although scientific and reporting integrity is expected for any study, quality assurance audit and review are rarely required for bioavailability, PK, and range-finding studies.

### Active pharmaceutical ingredient

API and DS are different terms for the same type of chemical entity used in the FDA *Guidance for Industry CGMP for Phase 1 Investigational Drugs *(2008) [3] and International Conference on Harmonisation (ICH) document *Q7: Good Manufacturing Practice Guide for Active Pharmaceutical Ingredients *(2000) [4] and, therefore, these terms are used interchangeably. According to the 2008 FDA guidance, both of these designations refer to 'any substance or mixture of substances intended to be used in the manufacture of a drug (medicinal) product and that, when used in the production of a drug, becomes an active ingredient of the drug product. Such substances are intended to furnish pharmacological activity or other direct effect in the diagnosis, cure, mitigation, treatment, or prevention of disease or to affect the structure and function of the body.' APIs include substances manufactured by processes such as chemical synthesis, fermentation, recombinant DNA or other biotechnology methods, isolation/recovery from natural sources, or any combination of these processes. Ultimately, the API must be well characterized in terms of structure identity (crystalline or polymorphic), counter ions (salts) and co-crystals, impurities, stability, chirality and enantiomer(s), appearance, solubility, and other chemical and physical properties. These properties will continue to be referenced throughout API scale-up process chemistry and GMP manufacturing.

All preclinical drug development programs require an adequate drug supply. As development progresses, increasing quantities of higher quality APIs are required for small non-good laboratory practice (non-GLP) efficacy studies, early formulation activities, *in vivo *dose range-finding studies, and finally rigorous IND-enabling GLP toxicology studies. For small molecule drugs, milligram quantities of research grade material are usually suitable for early stage *in vivo *efficacy and ADME studies, as well as small PK and dose range-finding studies (the latter may require gram quantities).

When a molecular entity is selected, preformulation activities commence to determine its physical and chemical properties, including counter ion salt or polymorphic form, solubility, and stability. The outcome of this stage is a recommended form, and the API portion of the project will transition to issues surrounding reaction efficiency, cost of goods, purity and control of impurities, and batch-to-batch consistency. In most cases the original medicinal chemistry reaction must be refined to improve availability of common starting materials and reaction reproducibility and scalability to maximize both product consistency and yield for each batch. As each batch is scaled up to produce greater quantities (grams to kilograms), the analytical control assays will require more stringent tolerance limits. Eventually these synthetic steps, along with control documentation, will be written into the master batch record and included in the IND package in the CMC section. In addition, API stability and degradation, including identity of major degradation products, will be evaluated for a variety of storage conditions and documented in the CMC section. These latter steps fall under GMP guidance and are beyond the capabilities of most investigative research laboratories. At some point in the process, the investigator may opt to transfer the synthetic process (along with appropriate legal intellectual property documentation) to a specialized contract research organization (CRO) that will produce required batches along with a Certificate of Analysis or GMP release for each batch. Once an API batch is released, it is ready to be used in GLP safety toxicology studies or prepared/formulated for clinical use.

### Formulation

Drug formulation, the mixing of API with other chemical ingredients to create the drug product (DP), is often a major hurdle in drug development. At this stage, the route of administration intended for the clinic should be clearly identified. Drugs may be introduced either enterally (oral, buccal, and rectal) or parenterally (not through the alimentary canal) including by injectable routes (intravenous, intramuscular, and subcutaneous), topically, and by inhalation. Except for imaging diagnostic agents, vaccines, and antibodies, most drugs targeting neurodegenerative diseases and their symptoms will be administered by the enteral or possibly topical routes. Drugs intended to be administered orally may be formulated as a solution, suspension, capsule, or tablet. Any formulation intended for human use is subject to rigorous quality control manufacturing and safety testing.

In addition to the clinical application, the specific physicochemical properties of a DS will influence formulation options. A number of parameters must be considered when creating the DP formulation. The components of any formulation must have physical and chemical compatibility with the DS. Patient factors to consider include tablet/capsule size, taste, stability and shelf life, and ease of use. Formulations may incorporate components such as dissociation enhancers that are found to improve bioavailability of the active DS. Solid formulations, particularly tablets, may be coated to improve swallowing, mask an unpleasant taste, protect ingredients during storage, improve appearance, and control drug release over time or target it to specific regions of the gastrointestinal tract.

In most instances, the clinical drug formulation is not fully optimized before submission of the IND and the initial first-in-human (FIH) clinical studies. Therefore, it is customary to compose a simple formulation to be presented as the DP in the IND and used in phase1 studies to deliver the drug to human subjects. For example, an oral FIH trial design may utilize drug supplied as a powder; an appropriate quantity is weighed out by the pharmacist and placed in a gelatin capsule prior to administration. For both oral and intravenous FIH trial designs, a specific amount of drug may be supplied in a clinical vial (also known as powder in bottle, or PIB) to which the appropriate volume of vehicle (specific liquid component) is added prior to administration. These approaches are most useful for early phase 1 trials involving relatively few human subjects. As the clinical trials become more complex and involve more subjects, oral formulations may be prepared in tablets or capsules containing scaled quantities, or 'strengths,' of the drug (for example, 50 mg, 100 mg, and 200 mg) so that each patient can take a combination of quantities to target individual body weight. Once the unit dose is established (usually after phase 2 and before phase 3 clinical trials), a formulation is selected as the final DP for later stage clinical trials and product release. As described above for APIs, all formulations intended for human use are prepared under rigorous specifications as outlined in the GMP guidelines.

### Analytical and bioanalytical methods

Starting from the initial drug discovery phase, analytical chemistry applications are found throughout the drug development process. These applications can be categorized into two major subdivisions: pharmaceutical analysis and bioanalysis. Pharmaceutical analysis involves the measurement of an analyte in a neat sample or formulation, whereas bioanalysis is the quantification of an analyte in a biological matrix.

#### Analytical methods

Reliable analytical methods are required to test and qualify in-coming materials, in-process methods, equipment, formulations, DSs, and DPs. These methods are critical for analyzing the various formulations that may be investigated for a final dosage form and are integral to quality control in GLP and GMP settings. In addition, FDA and ICH guidelines require stability testing on each lot of DS and DP. Therefore, analytical methods may need to be developed for a variety of materials and circumstances, each with a different intended purpose. For example, the analytical method required for formulation development may not require the same performance characteristics as those required for a stability-indicating method for DS or DP. Analytical work consists of two components: a research and development component and a GLP/GMP component. The research and development component includes analytical method development and analytical support for preformulation and formulation. The rest of the analytical work is conducted according to GLP and GMP guidelines. The quality control unit is responsible for the oversight of GMP analytical work.

It is essential that the validated methodology used to test the DS be used in clinical manufacturing. The developed method must satisfy two requirements: it must be accurate, requiring high specificity, good precision, and good reproducibility; and it should be practical, with the necessary ruggedness, sensitivity, and linearity. Assay methods are verified under the ICH guidelines for reproducibility, specificity, selectivity, accuracy, linearity, precision, applicable concentration range, limit of detection, limit of quantification, ruggedness, and robustness. The specifications for DS typically include a physiochemical characterization program that generally requires determination of the composition, physical properties, and primary structure of the desired product.

The suitability of a final compound for pharmaceutical use requires establishment of its identity and purity, as well as knowledge of its chemical and physical properties. Formulation analysis verifies the active and inactive components and dosage, assesses potency, determines shelf-life stability, confirms dissolution properties, and determines whether decomposition has occurred or impurities have been introduced during the formulation process. It is important to ensure that materials of known purity and defined quality are used in all studies and that they conform to applicable FDA regulatory requirements.

#### Bioanalytical methods

Physiologic fluids such as blood, plasma, and urine are analyzed to determine the fate and disposition of a DS administered to a test animal or patient. Aliquots of blood may be sampled over time to determine therapeutic drug concentration ranges. Often the goal is to assess the overall ADME characteristics of the DS. The concentration of the drug in the biological matrix changes with time, typically over a broad range, and, therefore, bioanalytical quantification limits are at concentrations much lower than those required for formulated or bulk drugs. An appropriate bioanalytical method is required to detect drug at these low levels, as well as linearly over an appropriate range. Matrix effects and stability issues can also make accurate analysis of the analyte difficult; these include, among many others, endogenous materials extracted from the biological matrix that may interfere in the analysis, enzymes in the biological fluid that are capable of metabolizing the analyte, plasma proteins that the analyte can bind to, and concomitant drugs that might interfere in the analysis. All these factors must be considered when planning an analysis.

### Pharmacokinetics, toxicokinetics, and metabolism

PK, or the study of the time course of a drug in the body incorporating the processes of ADME, is a key determinant in the selection of a viable drug candidate. Since many potential drugs are eliminated from further development because of poor PK properties, drug discovery programs now incorporate early ADME screens for desirable 'drug-like' properties to optimize the selection of successful candidates. These predictive ADME approaches include *in silico *models, physiochemical parameters, and *in vitro *assays of permeation and drug metabolism to better evaluate the properties of potential pharmaceuticals and to concentrate additional efforts on only the most promising compounds. After these screening data are evaluated in concert with efficacy results, compounds that are predicted to have favorable PK properties are studied further using *in vivo *animal models.

PK parameters are extrapolated from measurements of drug concentration in the plasma, blood, or other relevant biological matrix over a selected time period (usually several time points concluding at 24 or 48 hours post dose). PK provides information that can guide future animal and clinical studies for the selection of the dose levels and frequency of administration. The IND package requires PK data generated in two species (one rodent, one nonrodent), preferably using the same two species used for the safety studies. These studies usually include multiple dose levels so that PK dose dependency can be evaluated. Oral and intravenous administrations are compared to determine the oral bioavailability of the drug if an oral route is anticipated for the clinic. It is not necessary to delineate all ADME characteristics of a molecule at the time of IND submission, since the first clinical study generally focuses on the pharmacokinetics and metabolism of the drug in humans.

Toxicokinetic (TK) endpoints are similar to PK except that the samples are collected from animals during the toxicology and safety studies (described below) and usually do not cover as many time points. They help the investigator understand the drug's behavior at the maximal dose levels used in toxicology studies as well as steady state, accumulation, and trough levels after repeated administration. The same bioanalytical methods as developed for *in vivo *PK evaluations are usually suitable for measuring drug concentration in the relevant biological matrix (for example, plasma); however, the method will need to be validated if it is applied to GLP study samples.

Metabolism studies are also recommended by the FDA. These studies can be useful to evaluate the potential for drug-drug interactions and cytochrome P_450 _inhibition and to generate drug metabolite profiles for different species, including humans. Metabolism studies are typically conducted using *in vitro *methods for exposing hepatic microsomes, cytosolic fractions, hepatocyte cultures, or other applicable test systems to the drug. Using a bioanalytical method, any metabolites are described as a fractional percent of parent-drug peak. Any metabolite occurring at a significant level (for example, accounting for 10% or more of parent drug level, particularly when using test systems derived from primary human tissues) likely represents a significant metabolic pathway for the drug and may require further investigation, including elucidation of metabolite identity and biological activity. *In vitro *metabolism studies are frequently the first glimpse into the effects of human metabolic pathways on a drug and may reveal considerable metabolic differences between humans and preclinical species. In the event of significant interspecies metabolite profile differences, investigators may compare these with the human metabolite profile to identify which species, if any, most closely matches the humans, and then select this species for the pivotal safety and toxicology studies. Because of the importance of these interspecies results for understanding the potential disposition of the drug in humans, many development plans begin these studies about the same time as the first animal PK studies.

### Pivotal toxicology and safety

Despite numerous technical advances in the science of toxicology and attempts to develop *in silico *screening, the primary methods used to assess safety remain single- and repeat-dose toxicology studies conducted in rodent and nonrodent species. Definitive animal studies establish the safety characteristics, including the no observable adverse effect level (NOAEL), of the candidate drug. With very few exceptions, these studies are rigorously documented and conducted under regulatory guidelines – for example, FDA GLP. The highest dose levels tested in the definitive animal studies are almost always based on the maximum tolerated dose determined from the range-finding studies rather than either the expected dose level to be used in the clinical trial or an expected plasma concentration. By the time a drug reaches definitive animal safety testing, a human trials clinician is included in the drug development team to provide study details on the proposed FIH clinical trial. In general, the route and frequency of drug administration, vehicle, and dose formulation (if applicable) to be used in initial human trials is reflected in the definitive animal studies.

To account for differences between humans and laboratory species, a safety margin is established based on the NOAEL in the 'most sensitive' of the tested species. Toxicology studies are commonly conducted in rats and dogs, though other large animal species may be appropriate for specific products or therapeutic applications. For example, rabbits are frequently the species of choice for safety testing of vaccines. Preliminary toxicity studies are often performed as part of the lead compound selection process. For IND-directed safety studies, two complete GLP-compliant safety studies are generally required. The route of administration in these studies must be the same as the proposed clinical route. If the proposed route is oral, drug is administered by gavage to rats and by gavage or capsule to dogs. The duration of administration and dose regimen must, at a minimum, conform to the proposed clinical protocol. For example, if 14 days of continuous drug administration is proposed for the phase 1 clinical trial, then animal toxicity studies of at least 14 to 28 days are typically required to support a clinical study of this length. Although usually occurring after phase 1 dosing, longer term animal studies (for example, 60 days and longer) will be needed to support later stage human clinical trials. The frequency of dosing (for example, three times a week for 4 weeks) in the animal studies should also mimic the clinical dosing schedule.

Pivotal safety studies are performed with drug manufactured under GMP conditions whenever possible, although the FDA does not state this requirement. In the event that a drug is not manufactured under GMP conditions, the investigator is required to demonstrate that the clinical drug is essentially the same as that used in animal safety studies. If significant differences (for example, different counterion salts) are observed between GMP materials scheduled for the clinic and the materials used in a pivotal preclinical safety study, the regulating agency may deem the safety study to be invalid and request that bioequivalency studies be conducted or the pivotal study be repeated using the correct materials. One strategy used by some investigators to preemptively qualify some of the possible manufacturing impurities, such as enantiomers or side reactions, that may be present in batch production is to utilize a batch in the animal studies that contains at least 95% purity of the drug and up to 5% impurities. Because most repeat-dose toxicity studies of therapeutics reveal some adverse effects at higher dose levels, group assignment also includes a recovery group to evaluate whether adverse effects are transient or irreversible after repeat dosing. For dose level selection, allometric body surface area scaling may be used to convert from many preclinical species to human dose levels, with additional multiples added to account for interspecies differences and potential safety factors.

In addition to the pivotal safety studies, required ancillary studies, specified in ICH guidelines, complete most regulatory packages. Safety pharmacology studies are required regulatory elements outlined in ICH guideline S7A [5]. Standard procedures are established for the safety pharmacology core battery, which includes assessments for the central nervous system, cardiovascular system, and respiratory system. Genetic toxicology studies, outlined in ICH guidelines S2(R1) [6], are required for most small molecules. The FDA requires submission of data from three genetic toxicology assays for most IND applications: gene mutation in bacteria, including four strains of *Salmonella typhimurium *and one strain of *Escherichia coli *(Ames test); an *in vitro *mammalian cell assay (either evaluation of chromosomal damage in Chinese hamster ovary cells or the mouse lymphoma mutagenesis assay); and *in vivo *chromosomal damage in rat or mouse hematopoietic cells (such as the rodent bone marrow micronucleus assay). If the candidate drug may have immunosuppressive effects identified in earlier toxicity or safety studies, ICH guideline S8 [7] provides an overview of follow-up programs. Other studies that are frequently required in later stage drug submissions (after phase 1 clinical studies) include carcinogenicity and reproductive toxicology studies.

### Pre-IND meeting

Prior to the preparation of an IND application, it is recommended that the drug submitter request a pre-IND meeting. This meeting gives the drug submitter an opportunity to present the proposed preclinical and clinical trial protocols, as well as the proposed manufacturing and control testing of the DS and DP, to the appropriate FDA division representative(s). It also begins a dialog with the FDA to address specific questions concerning the proposed preclinical drug development program and its relation to the proposed early stage clinical trial(s). These discussions should occur prior to the initiation of complex preclinical activities (for example, GLP multiple-dose studies) to ensure that the design of the preclinical program addresses any potential concerns posed by the FDA. Incorporating this step into the process greatly improves the likelihood that the clinical trial will proceed within the planned timeframe following the IND submission. The pre-IND meeting request is accompanied by a background document authored by the submitter and sent to the reviewing agency that follows the FDA IND guidance. The submitter includes as much of the required information as possible by outlining completed, ongoing, and future drug development activities expected to occur prior to the clinical trial. Very importantly, the pre-IND document also includes a proposed agenda and a list of specific questions from the submitter to the FDA. The pre-IND materials include background information on the proposed use of the drug candidate and the results of the studies to determine its efficacy, summaries of the CMC process (including flowcharts), the proposed preclinical safety testing plan (current and future), the proposed phase I clinical trial(s) design, as well as any and all results collected to date. A response from the FDA is usually received within 10 working days following the meeting request, and if the FDA agrees to the request, the meeting usually occurs within 60 days of the submission. Following the pre-IND meeting, the preclinical development plan may be revised by the submitter to address any concerns expressed by the FDA.

### IND submission

The IND application submitted to the FDA describes all of the components in the development of the candidate drug. The details of the required content and format are described in detail in CFR title 21 part 312 [8] and FDA 'Guidance for Industry: Content and Format of Investigational New Drug Applications (INDs) for Phase 1 Studies of Drugs, Including Well-Characterized, Therapeutic, Biotechnology-derived Products' [9]. As per these guidance documents, the contents include: a cover sheet; a table of contents; an introductory statement and general investigational plan; an investigator's brochure – a compilation of the clinical and nonclinical data on the investigational product(s) that are relevant to the study of the product(s) in human subjects (ICH E6(R1) [10]); protocols – submission of a copy of the protocol for the conduct of each proposed clinical trial; chemistry, manufacturing, and control information; pharmacology and toxicology information; and previous human experience with the investigational drug.

The submission is formally identified using form FDA 1571, which provides details about the drug submitter and a checklist indicating the contents of the current FDA submission. Within 30 days of the IND submission, the FDA may put the clinical trial on hold or request additional data prior to the start of the study. If the submitter does not receive a reply within the 30-day timeframe, the trial may begin. At that time or prior to the start of the study, form FDA 1572 must be sent to the FDA. This form is completed by each clinical investigator at each clinical site and provided for submission to the FDA using the IND reference number. After the initial IND submission, the IND is updated through amendments, safety reports, or an annual report. Each update is accompanied by a form FDA 1571 identifying the subject of the current submission.

### Preclinical funding sources

**Figure 3:**
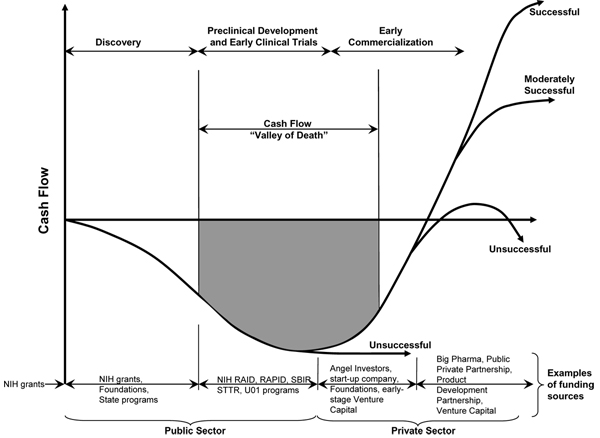
**Cash flow 'Valley of Death' diagram**. The cash flow 'Valley of Death' as a function of development stage (time) with typical funding sources at various stages (adapted from [12]). RAID, Rapid Access to Interventional Development; SBIR, Small Business Innovative Research; RAPID, Rapid Access to Preventive Intervention Development; STTR, Small Business Technology Transfer.

The transition from research and preclinical development to the clinic is so perilous that it is frequently referred to as the 'Valley of Death' [11,12] (Figure 3). Funding challenges contribute to this bleak landscape. Successful transitioning often requires a combination of both private and public sector investments targeting the most promising drug candidates. Several public and private sources are available to provide funding for preclinical activities (Table 3)

**Table 3:** Preclinical development funding links

**NIH programs**	
RAID pilot program	
SBIR/STTR	
	
**Alzheimer's disease**	
Alzheimer's Drug Discovery Foundation	
	
**Amyotropic lateral sclerosis**	
Amyotropic Lateral Sclerosis Association	
	
**Huntington's disease**	
CHDI, Inc.	
High Q Foundation	
	
**Multiple sclerosis**	
National Multiple Sclerosis Society	
Myelin Repair Foundation	
	
**Parkinson's disease**	
Michael J Fox Foundation for Parkinson's Research	

RAID, Rapid Access to Interventional Development; SBIR, Small Business Innovative Research; STTR, Small Business Technology Transfer.

As part of its Roadmap for Medical Research Initiative, the US Government provides resources for nonprofit and commercial institutions through the NIH Rapid Access to Interventional Development (RAID) pilot program and the Small Business Innovative Research (SBIR) and Small Business Technology Transfer (STTR) programs. The RAID program is an NIH Roadmap initiative modeled after the National Cancer Institute (NCI) RAID program. The RAID programs are not grant-equivalent funding sources. Rather, they provide access to NIH contract resources to support preclinical development of small molecule drug candidates. Potential services may include bulk API synthesis and supply; GMP manufacturing of clinical supply; formulation development; analytical assays; and development and validation of animal PK, toxicology, and safety tests. Currently, the RAID program does not fund development of biologics, *in vitro *or *in vivo *efficacy testing, or mechanism-of-action studies. Support for contracted preclinical activities comes from the budgets of the NIH Roadmap as well as the sponsoring NIH institute. This program is currently available to investigators at academic, nonprofit, and SBIR-eligible (see below) institutions. Additional information is available at the NIH RAID program web site [13] and from program officers at appropriate NIH institutions.

Although RAID support is limited to developing small molecules, other promising approaches such as biologics, vaccines, antibodies, and other non-small-molecule therapies have access to preclinical PK and toxicology testing services through STTR, SBIR, and institute-sponsored programs. The SBIR and STTR programs (R43, R44, R41, and R42) are designated for domestic small (<500 employees) businesses to support commercialization of discoveries and inventions. Eligibility is currently restricted to companies that are at least 51% owned by one or more individuals who are US citizens or permanent resident aliens. In the SBIR program, the primary employer of the Principal Investigator must be an established small business. In contrast, the STTR funds partnerships between an academic or nonprofit institution and a small company without restrictions on the employer of the Principal Investigator. Phase I SBIR and STTR programs are intended to test the technical feasibility and commercialization potential of proposed research and development projects. Phase II awardees receive further funding and time to develop the lead, although the funds are usually not adequate to support a full preclinical program. In early 2008, the NIH offered phase II SBIR awardees an additional opportunity to apply to the Manufacturing Assistance Program for help in overcoming manufacturing issues.

In addition to the above NIH funding resources, many NIH institutes offer funding through 'Cooperative Agreement Research Project Awards' (U01) to academic, not-for-profit, and other entities. These awards support a discrete, specified, circumscribed research project to be performed by the named investigators in cooperation with NIH program staff and are milestone driven for continued funding to reach an IND filing.

In addition to government-funded programs, public and private foundations offer funding, outsourcing, and other resources for translational development [14,15]. Table 3 lists web links for several foundations that support preclinical development for neurodegenerative diseases. The Alzheimer's Drug Discovery Foundation (ADDF) was established in 2004 by the Institute for the Study of Aging to support drug discovery research for Alzheimer's and related diseases. ADDF preclinical support includes funding for drug discovery and development and sponsorship of forums to share best practices. The Amyotropic Lateral Sclerosis Association launched the Translational Research Advancing Therapy for Amyotropic Lateral Sclerosis (TREAT ALS) initiative to accelerate drug development and support clinical trials. To support rapid development of treatments for multiple sclerosis, the National Multiple Sclerosis Society founded Fast Forward in 2007 as a wholly owned nonprofit subsidiary. Its purpose is to fund preclinical studies leading to clinical trials, support startup and existing companies, and repurpose existing drugs as potential multiple sclerosis therapeutics. The Myelin Repair Foundation supports drug development focused on re-myelination of multiple sclerosis lesions and has organized a consortium of leading labs from multiple universities to collaborate on therapies that repair damaged myelin. The High Q Foundation established CHDI Foundation, Inc. as a virtual biotechnology company to discover and develop drugs targeting Huntington's disease. The Michael J Fox Foundation for Parkinson's Research (MJFF) supports drug development through several funding mechanisms, including Rapid Response Innovation Awards with a rolling application to support novel research proposals, a Target Validation program, Novel Approaches to Parkinson's Disease Drug Discovery in partnership with Elan Pharmaceuticals, and the LEAPS Initiative to support a team approach to major developmental challenges. The MJFF Therapeutics Development Initiative exclusively funds biotechnology and pharmaceutical companies to support drug and biomarker development. This partial list of foundation initiatives illustrates the variety and strength of support for preclinical development available to fill critical gaps in expertise and financial resources.

### Outsourcing

Unlike large pharmaceutical companies, most academic laboratories and small or virtual start-up companies lack the facilities, expertise, and regulatory oversight to complete the preclinical development testing required to file an IND. These organizations frequently employ the services offered by preclinical CROs. Under a contract with the drug submitter, preclinical CROs, such as SRI International, Covance, and Charles River Laboratories, will perform individual tasks and studies or offer a full development package. Some CROs have contracts with NIH institutions (for example, the National Institute on Aging (NIA)) to perform specific preclinical tasks for a submitting investigator in response to a request from the NIH Project Officer. In other cases, a CRO may be included as a subcontractor under an SBIR grant or as a designated partner under a foundation grant.

## Conclusion

Drug development through IND submission requires effective communication and execution by a team with a diverse skill set. The progressive transition of a drug candidate from early to late preclinical stages is particularly critical. Team members need to understand the intended clinical plan for a drug candidate and anticipate potential problems to execute an effective preclinical strategy. The TPP provides a framework for defining the desired features of the new DP, known or suspected risks and liabilities, and metrics of success.

Despite careful planning, most drug candidates fail. Reasons for failure include poor solubility, life threatening or other undesirable side effects, poor biodistribution by the proposed clinical route of administration, prohibitive scale-up and manufacturing costs, market competition, and poor efficacy in early clinical trials. The best that any project team can do is set clear 'Go/No-Go' criteria and design a preclinical strategy focused on identification of key issues to weed out problematic drug candidates early in the process. Preclinical drug development is often called the 'Valley of Death,' where good ideas often die through design flaws, lack of specialized expertise, and insufficient funding. Increasingly, public and private organizations are coming to the aid of investigators with outsourcing and funding support (Table 3).

The 'Graying of America' [16] is upon us. Treating the growing number of patients afflicted with neurodegenerative diseases threatens the resources of our health care system, the financial stability of our country, and the quality of life for patients and their caregivers. More than ever, effective navigation through the path of preclinical development is essential for the pharmaceutical industry and for society.

## List of abbreviations used

ADDF: Alzheimer's Drug Discovery Foundation; ADME: absorption, distribution, metabolism, and excretion; API: active pharmaceutical ingredient; CFR: Code of Federal Regulations; CMC: chemistry, manufacturing, and controls; CRO: contract research organization; DP: drug product; DS: drug substance; FDA: US Food and Drug Administration; FIH: first-in-human; GLP: good laboratory practice; GMP: good manufacturing practice; ICH: International Conference on Harmonisation; IND: Investigational New Drug; MJFF: Michael J Fox Foundation for Parkinson's Research; NOAEL: no observable adverse effect level; PK: pharmacokinetics; RAID: Rapid Access to Interventional Development; SBIR: Small Business Innovative Research; STTR: Small Business Technology Transfer; TPP: target product profile.

## Competing interests

KLS and EGS are employed by SRI International.

## Authors' contributions

KLS and EGS participated in the drafting and editing of the manuscript. Both authors read and approved the final manuscript
